# MTFR2, A Potential Biomarker for Prognosis and Immune Infiltrates, Promotes Progression of Gastric Cancer Based on Bioinformatics Analysis and Experiments

**DOI:** 10.7150/jca.58158

**Published:** 2021-04-26

**Authors:** Hai Zhu, Gang Wang, Haixing Zhu, Aman Xu

**Affiliations:** 1 Department of General Surgery, The First Affiliated Hospital of Anhui Medical University, Hefei 230001, People's Republic of China.; 2 Department of General Surgery, The Fourth Affiliated Hospital of Anhui Medical University, Hefei 230001, People's Republic of China.; 3 Department of Gastrointestinal Surgery, The First Affiliated Hospital of USTC, Division of Life Sciences and Medicine, University of Science and Technology of China, Hefei 230031, People's Republic of China.

**Keywords:** MTFR2, Gastric cancer, Mitochondria, Proliferation, Prognosis, Immune.

## Abstract

**Background:** Mitochondrial fission regulator 2 (MTFR2) which can promote mitochondrial fission, has recently been reported to be involved in tumorigenesis. However, little is known about its expression levels and function in gastric cancer (GC). This study aims to clarify the role of MTFR2 in GC.

**Methods:**We firstly determined the expression level and prognostic value of MTFR2 in GC by integrated bioinformatics (Oncomine, GEPIA, Kaplan-Meier Plotter database) and experimental approaches (RT-qPCR, western blot, immunohistochemistry). After constructing stable down-regulated GC cells, the biological functions of MTFR2 *in vitro* and* in vivo* were studied through cell clone formation, wound healing, transwell and tumor formation experiments.To understand the reason for the high expression of MTFR2 in GC, copy number alternation, promoter methylation and mutation of MTFR2 were detected by UALCAN and cBioPortal. TargetScanHuman and PROMO databases were also used to explore the miRNAs and transcription factors of MTFR2, and the regulatory network was visualized by Cytoscape. LinkedOmics was used to detect the co-expression profile, and then these co-expressed genes were used for gene oncology function and pathway enrichment analysis to deepen the understanding of MTFR2 mechanism. The protein interaction network of MTFR2 was constructed by the GeneMANIA platform. Docking study of the binding mode was conducted by H DOCK webserver, and PYMOL is used for visualization, and analysis. TIMER database was used to explore the correlation between MTFR2 expression level and immune cells infiltration and gene markers of tumor infiltrating immune cells.

**Results:** We demonstrated that MTFR2 was up-regulated in GC, and its overexpression led to poorer prognosis. MTFR2 downregulation inhibited the proliferation, migration, and invasion of GC cells *in vitro* and* in vivo*. By bioinformatics analysis, we identified the possible factors in MTFR2 overexpression. Moreover, function and pathway enrichment analyses found that MTFR2 was involved in chromosome segregation, catalytic activity, cell cycle, and ribonucleic acid transport. A MTFR2-protein interaction network revealed a potential direct protein interaction between MTFR2 and protein kinase adenosine-monophosphate-activated catalytic subunit alpha 1 (PRKAA1), and their potential binding site was predicted in a molecular docking model. In addition, we also found that MTFR2 may be correlated with immune infiltration in GC.

**Conclusions:** Our study has effectively revealed the expression, prognostic value, potential functional networks, protein interactions and immune infiltration of MTFR2 in GC. Altogether, our data identify the possible underlying mechanisms of MTFR2 and suggest that MTFR2 may be a prognostic biomarker and therapeutic target in GC.

## Introduction

Gastric cancer (GC) is a common lethal malignancy. In 2018, its incidence and mortality were ranked fifth and third, respectively, worldwide 1. Although its prognosis has increased with current treatment methods, the overall five-year survival rate of GC patients is still low 2. The poor prognosis is mainly due to limited treatment options, especially in patients resistant to chemotherapy 3. In the past decade, research on mitochondrial metabolism has greatly expanded the understanding of cancer biology and treatment development 4.

Mitochondrial fission and fusion regulates mitochondrial morphology, location, and transport, thereby playing vital roles in maintaining normal cellular function, including cellular metabolism, the generation of reactive oxygen species, sulfide oxidation, cellular signaling, calcium balance, lipid modification, biosynthetic metabolism, and cell death 5-8. Accordingly, abnormal mitochondrial fission can lead to a series of intracellular metabolic responses and, ultimately, dysfunction 9. Mitochondrial Fission Regulator 2 (MTFR2), also known as family with sequence similarity 54 member A, regulates mitochondrial fission 10, 11. Recent studies reported that its aberrant expression promotes the proliferation, migration, and invasion of tumor cells and correlates with breast cancer and oral squamous carcinoma prognosis 12, 13. However, currently available data on MTFR2 are insufficient in describing its role in tumor initiation and development. Therefore, we aimed to explore its expression and function in GC.

In this study, we systematically investigated the expression level, prognostic value, potential function, regulatory mechanisms, and immune infiltrates of MTFR2 in GC using bioinformatics analyses and experimental approaches. Altogether, the results provide evidence that MTFR2 can be a potential prognostic biomarker and therapeutic target in GC.

## Materials and methods

### Public database analysis

The ONCOMINE (https://www.oncomine.org) 14, 15, GEPIA2 (http://gepia2.cancer-pku.cn) 16, 17, and GENT (http://medicalgenome.kribb.re.kr/GENT) 18 databases were used to compare MTFR2 messenger ribonucleic acid (mRNA) expression between tumors and normal tissue among various types of cancers, especially GC. Representative immunohistochemistry (IHC) images from the Human Protein Atlas (HPA) (https://www.proteinatlas.org) 19-21 database were examined to detect MTFR2 subcellular localization and tissue expression abundance in cancerous and normal tissues. Next, the Kaplan-Meier Plotter (https://kmplot.com) 22 was used to evaluate the prognostic value of MTFR2 in GC.

To better understand the mechanisms underlying MTFR2 overexpression, MTFR2 promoter methylation levels were investigated using UALCAN (http://ualcan.path.uab.edu), while mutations and copy number alterations (CNAs) were quantified using cBioportal (http://www.cbioportal.org)^23^, ^24^. TargetScanHuman^25^ and PROMO^26^ were employed to identify MTFR2 micro ribonucleic acids (miRNAs) and transcription factors, with the results visualized via Cytoscape^27^.

The LinkedOmics (http://www.linkedomics.org) 28 database was mined to find MTFR2 co-expressed genes based on Pearson's correlation coefficient, with the results displayed via volcano plots and heat maps. The linear regressions between MTFR2 and its top three positive or negative genes were confirmed using GEPIA2. In addition, the enrichment function of Gene Ontology (GO) annotations and Kyoto Encyclopedia of Genes and Genomes (KEGG) pathways was conducted using LinkedOmics (http://www.linkedomics.org). LinkedOmics was also searched via gene set enrichment analysis (GSEA) to explore kinase-, miRNA-, and transcription factor-target enrichment. Finally, GeneMANIA (http://genemania.org) was applied to obtain and construct a MTFR2 gene interaction network 29.

TIMER (https://cistrome.shinyapps.io/timer) database is a publicly available comprehensive resource for systematic analysis of tumor immune-infiltrates 30. The gene modules were used to explore the correlation between MTFR2 expression level and immune cells infiltration, including B cells, CD4 + T cells, CD8 + T cells, neutrophils, macrophages and dendritic cells in GC. The correlation module was used to analyze the relationship between the gene markers of tumor infiltrating immune cells and MTFR2 expression with or without tumor purity adjustment. The correlation coefficient is estimated using Spearman's correlation method.

### Clinical specimens

All tissue samples used for IHC and western blot were collected from patients diagnosed with gastric cancer at the First Affiliated Hospital of Anhui Medical University. The current research work has been approved by the Academic Committee of The First Affiliated Hospital of Anhui Medical University and was conducted in line with the rules put forward in the Declaration of Helsinki. The retrieval of each and every dataset was carried out from the publishing literature, accordingly confirming that all of the written informed consents were attained.

### Cell culture and stable transfection

A normal gastric mucosal cell line (GES1) and four human gastric cell lines (MKN45, MGC803, AGS, MKN28) were obtained from the Cell Bank of the Chinese Academy of Sciences (Shanghai, China). All cells were cultured in RPMI-1640 or DMEM (BI, Haemek, Israel) supplied with 10% fetal bovine serum (BI, Haemek, Israel) and 1% penicillin- streptomycin (Gibco; Thermo Fisher Scientific Inc., Waltham, MA, USA) in an atmosphere containing 5% CO_2_ at 37˚C. They were authenticated by STR profiling and tested to be pathogen and mycoplasma negative before the experiments (Biowing Biotechnology, Shanghai, China). sh1-MTFR2, sh2-MTFR2 and sh-NC were synthesized by GenePharma Co. Ltd (Suzhou, China), the sequence are as follows: sh1-MTFR2: 5′-CCGGGCAATTGTGGAAATGCAGGAACTCGAGTTCCTGCATTTCCACAATTGCTTTTTTG-3′; sh2-MTFR2: 5′- CCGGGTGGATCTATGGTTCCATCTTCTCGAGAAGATGGAACCATAGATCCACTTTTTTG-3′. Lipofectamine 3000 (Invitrogen; Thermo Fisher Scientific Inc., Waltham, MA, USA) was used to perform the transfection according to the manufacturer's guidelines.

### Immunohistochemistry staining

All of the collected tissue specimens were conducted to formalin fixation and embedded with paraffin, 4μm thick tissue sections were carried out to immunohistochemistry staining. The IHC experimental method is the same as that reported in our previous literature 31. The intensity score was analyzed as follows: 0: no staining, 1: weak staining; 2: medium staining, and 3: strong staining. The proportion score was further classified as 0: 0%; 1: 1-25%, 2: 26-50%, 3: 51-75%, 4: >75%. The immunoreactive score was calculated by multiplying the intensity score and proportion score. For statistics analysis, scores of 0 to 7 were considered low expression and scores of 8 to 12 were considered high expression. Two independent pathologists accompanied and assessed the staining procedure and results.

### Reverse transcription‐quantitative PCR (RT‐qPCR)

Following lentiviral transfection and establishment of stable cell lines, total RNA was extracted from cells by 1.0 ml TRIzol. The total RNA was reverse transcribed to cDNA by PrimeScriptTM RT Master Mix (Takara, Japan). RT-PCR was performed using the Applied Biosystems 7500 Fast Real-Time PCR System with TB GreenTM Premix Ex TaqTM II (Takara, Japan) to detect MTFR2 mRNA. GAPDH was amplified in parallel as an internal control. MTFR2 expression was calculated using the 2^-∆∆Ct^ method. The following primers were used: MTFR2:forward:5′-ATTTTGGCGTTCCTGTAGAACA-3′; reverse:5′- CAGAGTTCAAGAGCGGGATCA′.

### Western blot

Radioimmunoprecipitation assay buffer (Beyotime Biotechnology, China) was used to extract total protein from gastric cancer tissue samples and cells, supernatants were collected for Western blot assays. Protein concentration was determined using a bicinchoninic acid (BCA) protein assay kit (CW0014S, CWBIO, China). Total protein samples were subjected to 10% sodium dodecyl sulphate-polyacrylamide gel electrophoresis (SDS-PAGE) and transferred to polyvinylidene difluoride membranes. After blocking with 5% bovine serum albumin, the membranes were incubated with anti-MTFR2 (NBP1-84967, 1:1000, Novus Biologicals, USA); anti-β-Tubulin (ab6406, 1:5000, Abcam, England) at 4°C overnight. After extensive washing, the membrane was incubated with the secondary antibody at room temperature for 1 h and was then exposed to an enhanced chemiluminescence reagent (Millipore, USA).

### Cell clone formation

Approximately 2.0×10^2^ cells per well were grown in 6-well plates containing DMEM medium with 10% FBS at 37°C. After two weeks, the cells were treated with 10% formaldehyde solution and stained with 0.1% crystal violet (Sigma, USA). The calculation of the colonies formed were performed using a light camera without magnification.

### Wound healing assay

Approximately 4.0×10^5^ cells were seeded in a 6-well plate and cultured overnight at 37°C in an atmosphere containing 5% CO_2_ to achieve >80% confluence. Subsequently, a 200 μl pipette tip was used to scrape longitudinally in the center of the bottom of the sample well. The separated cells were washed away with PBS, and then serum-free medium was added. Images were taken at 0 and 48 hours after the wound, and the healing was checked using an optical microscope (IX81, Olympus, Japan) at 40 × magnification.

### Transwell assay

A total of 5 × 10^4^ cells was resuspended in 250 µL of corresponding plain medium in the upper chamber (cat. no. 354480, BD Biosciences, Franklin Lakes, New Jersey, USA) while the lower chambers were filled with 750 µL of corresponding complete medium. After incubating for 24h at incubator, the upper chambers were fixed with 100% ice methanol for 10 min and stained with 0.1% crystal violet at room temperature. The number of transmembrane cells was calculated under a microscope (IX81, Olympus, Japan) at 100 × magnification.

### Tumor formation assay

BALB/c nude mice (male, 6 weeks and ~18.30g) were purchased from the Institute for Experimental Animals of the Chinese Academy of Medical Sciences (Beijing, China). MGC803 cells (2.0×10^6^) with sh-NC or sh1-MTFR2 were subcutaneously injected into the shoulder. The nude mice are maintained under pathogen-free conditions and eat freely at 20-26°C, 40-70% humidity and 12/12 light/dark cycle. After 4 weeks, the mice were sacrificed their tumors were excised. The animal experiment was approved by the Ethics Committee of Anhui Medical University.

### Molecular docking analysis

The structure of protein kinase adenosine-monophosphate-activated catalytic subunit alpha 1 (PRKAA1) is obtained from PDB ID: 6C9H, chain A. The MTFR2 structure is conducted through homology modeling using the MPI Bioinformatics Toolkit webserver. (Accession number of the protein sequence used: NP_001092756.1). Docking study of the binding mode between MTFR2 and PRKAA1 was conducted by H DOCK webserver, and PYMOL is used for visualization, analysis, and mapping.

### Statistical methods

All of the statistical analyses were carried out using SPSS 21.0 software (IBM Corp; Armonk, NY). The statistical analysis between two variables was performed by Student's t-test, and one-way ANOVA analysis was performed for more than two variables. Survival curves and relative results generated from Kaplan-Meier Plotter database was shown with hazard ratio (HR) and *P*-*value* from a log-rank test. *P-value* < 0.05 was considered as statistically significant.

## Results

### MTFR2 expression and prognostic value in GC

**Figure 1:**
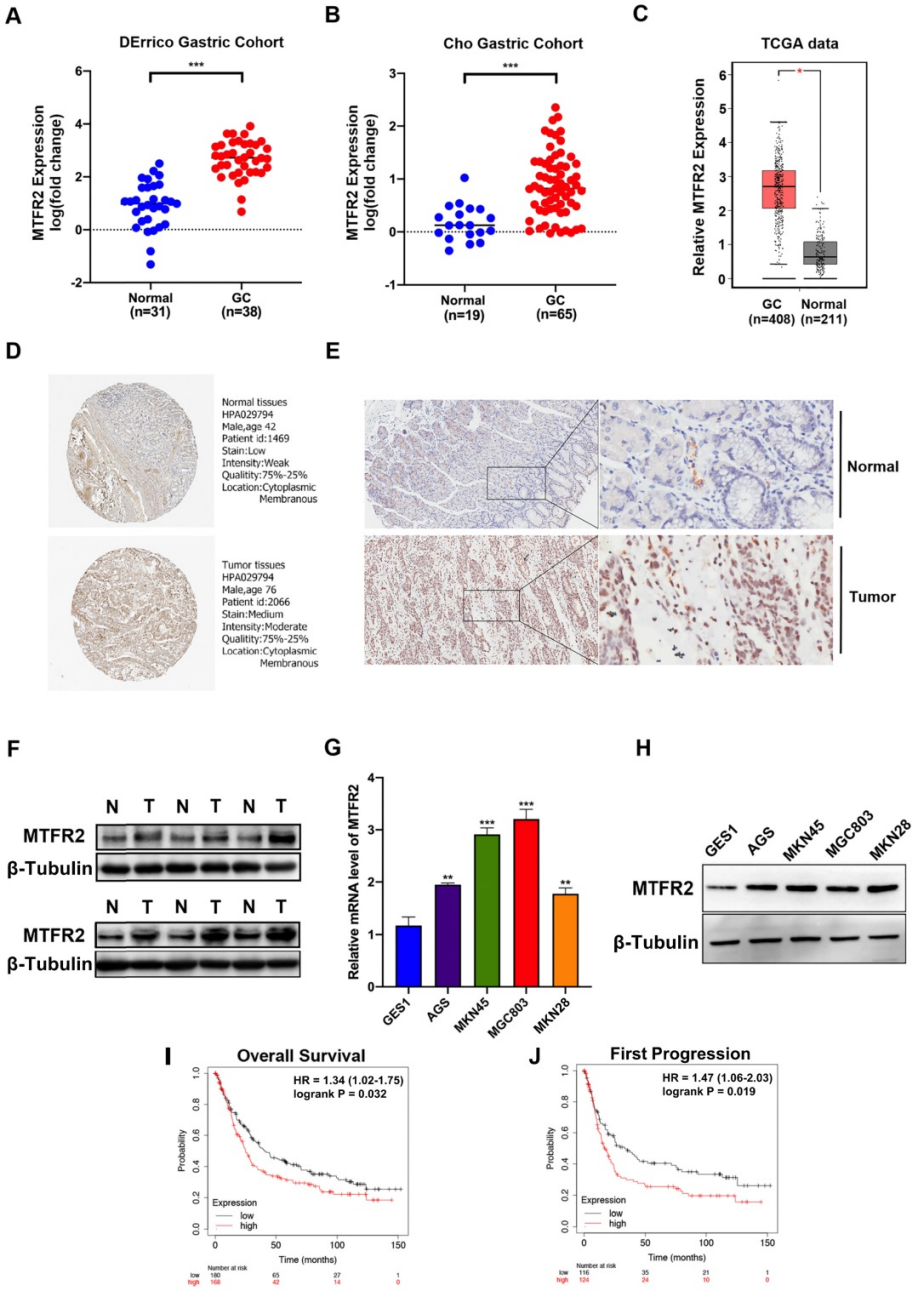
MTFR2 is highly expressed and correlated with poor prognosis of patients in GC. (A-B) The mRNA expression level of MTFR2 between GC and normal tissues in DErrico and Cho Gastric Cohort from Oncomine (***P < 0.001). (C) The mRNA expression level of MTFR2 between GC and normal tissues in TCGA from GEPIA (*P < 0.05). (D) Representative IHC staining images of MTFR2 expression in normal and tumor tissues from HPA. (E) Representative IHC staining images of MTFR2 expression in normal and tumor tissues. (F) MTFR2 protein expression in GC tissues; (G-H) MTFR2 mRNA and protein expression in gastric cell and gastric cancer cell lines (**P < 0.01, ***P < 0.001). (I-J) GC patients with a high level of MTFR2 showed worse OS and FP than those with a low level of MTFR2 (HR:hazard ratio).

Three databases (ONCOMINE, GEPIA2, and GENT) were mined to analyze MTFR2 expression levels among different cancers (Fig. S1A-C). In all three databases, MTFR2 mRNA expression in gastric, breast, colon, cervical, ovarian, and pancreatic cancer, was significantly higher than that in normal tissue (Fig. S1D). Subsequently, two GC datasets, DErrico and Cho from ONCOMINE, were used to quantify differences in MTFR2 mRNA expression between GC and normal tissues, with the results showing that MTFR2 mRNA expression was significantly higher in GC patients (Fig. 1A-B). Data from the TCGA database also supports this finding (Fig. 1C). We qualitatively evaluated MTFR2 subcellular localization and tissue expression abundance via the Human Protein Atlas (Fig. 1D). Western blot and IHC data indicated that MTFR2 protein expression was higher in GC tissue compared to adjacent normal tissue (Fig. 1E-F). Moreover, MTFR2 expression, as assayed by RT-qPCR and western blot in GC cell lines (AGS, MKN45, MGC803, and MKN28), was significantly higher than in normal gastric mucosal cell line (GES1) (Fig. 1G-H).

The Kaplan-Meier Plotter tool, via high-throughput analysis and detailed clinical prognosis data, can characterize and predict the relationship between MTFR2 mRNA levels and GC patient survival. Patient overall survival (OS) and first progression rates were both significantly negatively correlated with MTFR2 expression (Fig. 1I-J). These results indicate that MTFR2 is highly expressed in GC and suggest that it is vital in tumorigenesis.

### The biological function of MTFR2 as assayed *in vitro* and* in vivo*

**Figure 2:**
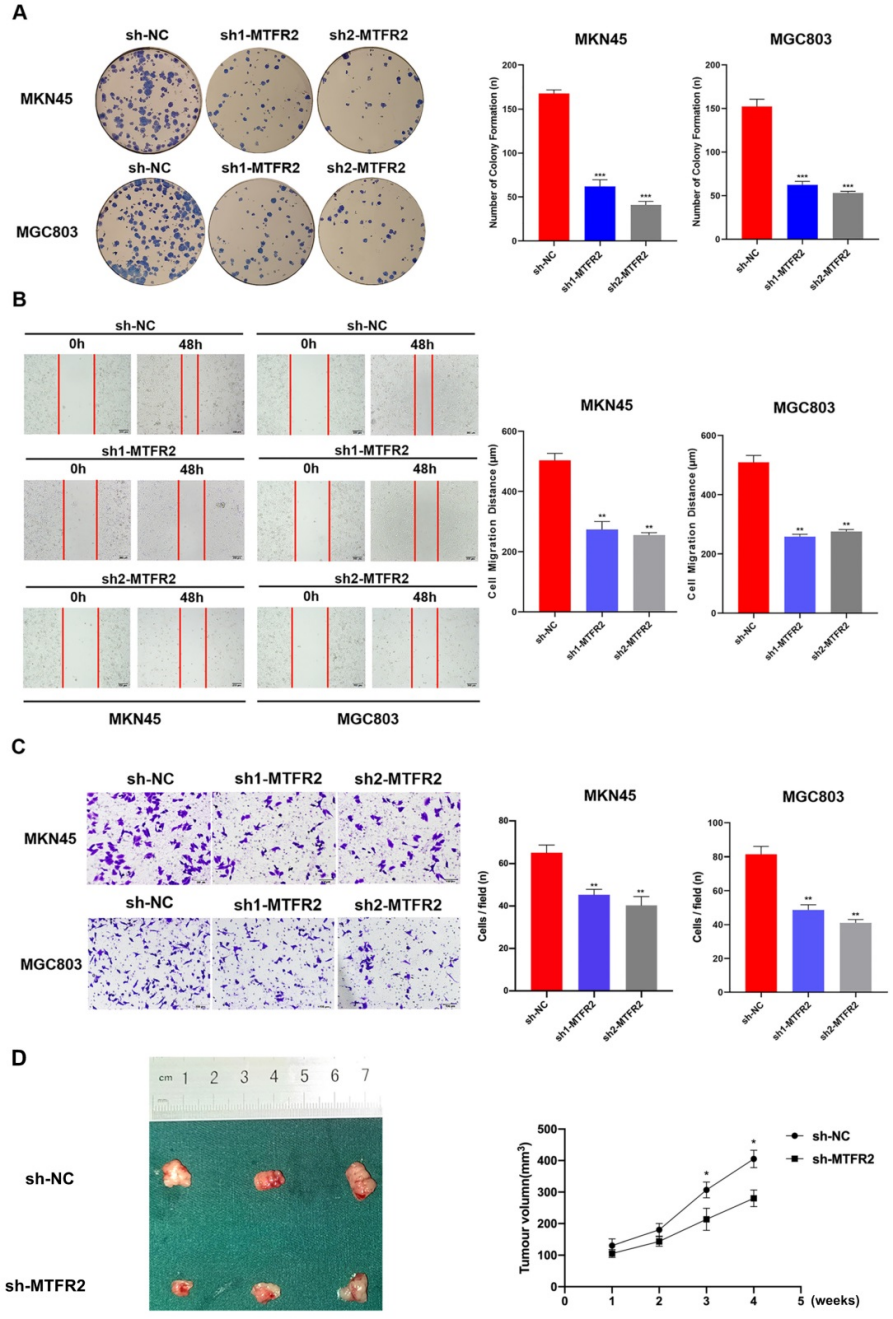
** Tumor-promotive effects of MTFR2 in GC cells.** (A). Representative images of colony formation induced by sh-NC, sh1-MTFR2, sh2-MTFR2 in MKN45 and MGC803 cell lines. The numbers of colonies were measured and are shown in the bar graph. All data were mean ± SD and from three independent experiments (***P < 0.001). (B). A cell wound-healing assay showed that cell motility was decreased after MTFR2 knockdown in the MKN45 and MGC803 cell lines. Microscopic images were acquired at 0 and 48 h (magnification, ×40). The migratory distance of the cells was measured and are shown in the bar graph. All data were mean ± SD and from three independent experiments. (**P < 0.01). (C). Cell invasion assays of sh-NC, sh1-MTFR2 and sh2-MTFR2 in MKN45 and MGC803 cell lines, Invaded cells were fixed and stained with crystal violet (magnification, ×100). The number of invaded cells was calculated and is shown in the bar graph. All data were mean ± SD and from three independent experiments (**P < 0.01). (D). Xenograft tumor experiments in nude mice proved that knockdown of MTFR2 reduces tumorigenesis ability. Representative images of nude mouse xenograft tumors. Statistical analysis of xenograft tumour sizes revealed that tumour growth was markedly inhibited by MTFR2 silencing (*P < 0.05).

To study the biological function of MTFR2 in GC, we down-regulated its expression by constructing MTFR2 short hairpin ribonucleic acid (shRNA)-containing lentiviruses, sh1-MTFR or sh2-MTFR2. MTFR2 knockdown efficiency was confirmed via western blot and RT-qPCR (Fig. S2). The plate colony formation assay was used to examine the impact of MTFR2 on GC cell proliferation; following sh-MTFR2 transfection, colony formation activity was inhibited (Fig. 2A). Subsequently, we performed wound healing and transwell assays to explore the role of MTFR2 in cell migration and invasion; MTFR2 knockdown significantly suppressed cell migration and invasion capability *in vitro* (Fig. 2B-C). To further validate the effect of MTFR2 on tumor *in vivo*, the nude mice xenograft tumor model was constructed. The results indicated that cells with down-regulated MTFR2 had a significantly reduced ability of inducing tumor formation compared with the controls (Fig. 2D).

### The regulatorory network of MTFR2 in GC

**Figure 3:**
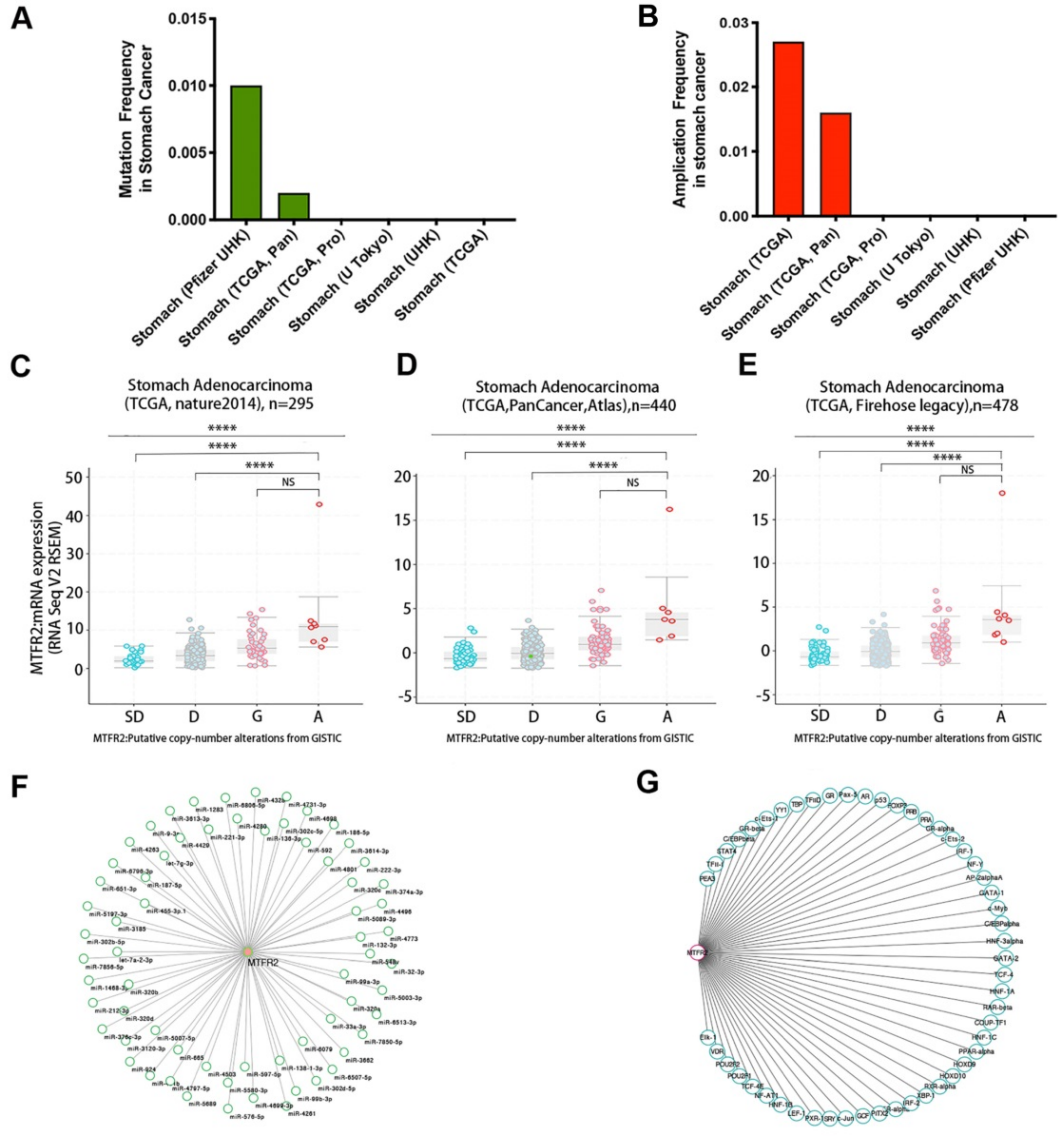
** Regulatory network of the expression of MTFR2 in GC.** (A-B) MTFR2 mutation and amplication frequency in gastric cancer was presented as bar diagram; (C-E) The graph depicts the correlation between MTFR2 expression and copy number alterations in gastric cancer of TCGA data. (Abbreviations: deep deletions = DD, shallow deletion = SD, diploid = D, gain = G, and amplification =A; NS:not significance, ****p<0.0001). (F-G) miRNAs and transcription factors network of MTFR2.

Gene expression levels are controlled by promoter methylation, with lower levels of methylation associated with increased mRNA expression of the target gene. Therefore, we used UALCAN to quantify MTFR2 promoter methylation in GC. We found that methylation levels of the MTFR2 promoter in tumors did not significantly differ compared to their normal tissue counterparts (Fig. S3A). The cBioportal database was mined to analyze MTFR2 gene mutations and CNA status. The results revealed that MTFR2 possessed two mutation sites (Fig. S3B). The mutation rate was 1% and 0.2%, respectively, in the Pfizer UHK and TCGA Pan datasets, and no mutations were detected in the four other datasets (Fig. 3A). With respect to MTFR2 CNAs, no gene depletion was detected in any dataset. The dominant alteration was amplification, and it was detected in approximately 1.6-2.7% of patients (Fig. 3B). In GC, amplification was significantly correlated with MTFR2 expression levels (one-way analysis of variance,* p* < 0.0001; Fig. 3C-E). Moreover, 66 miRNAs and 51 transcription factors of MTFR2 were identified in the TargetScanHuman (Fig. 3F) and PROMO databases (Fig. 3G), respectively.

### MTFR2 co-expression profiles and enrichment analysis in GC

**Figure 4:**
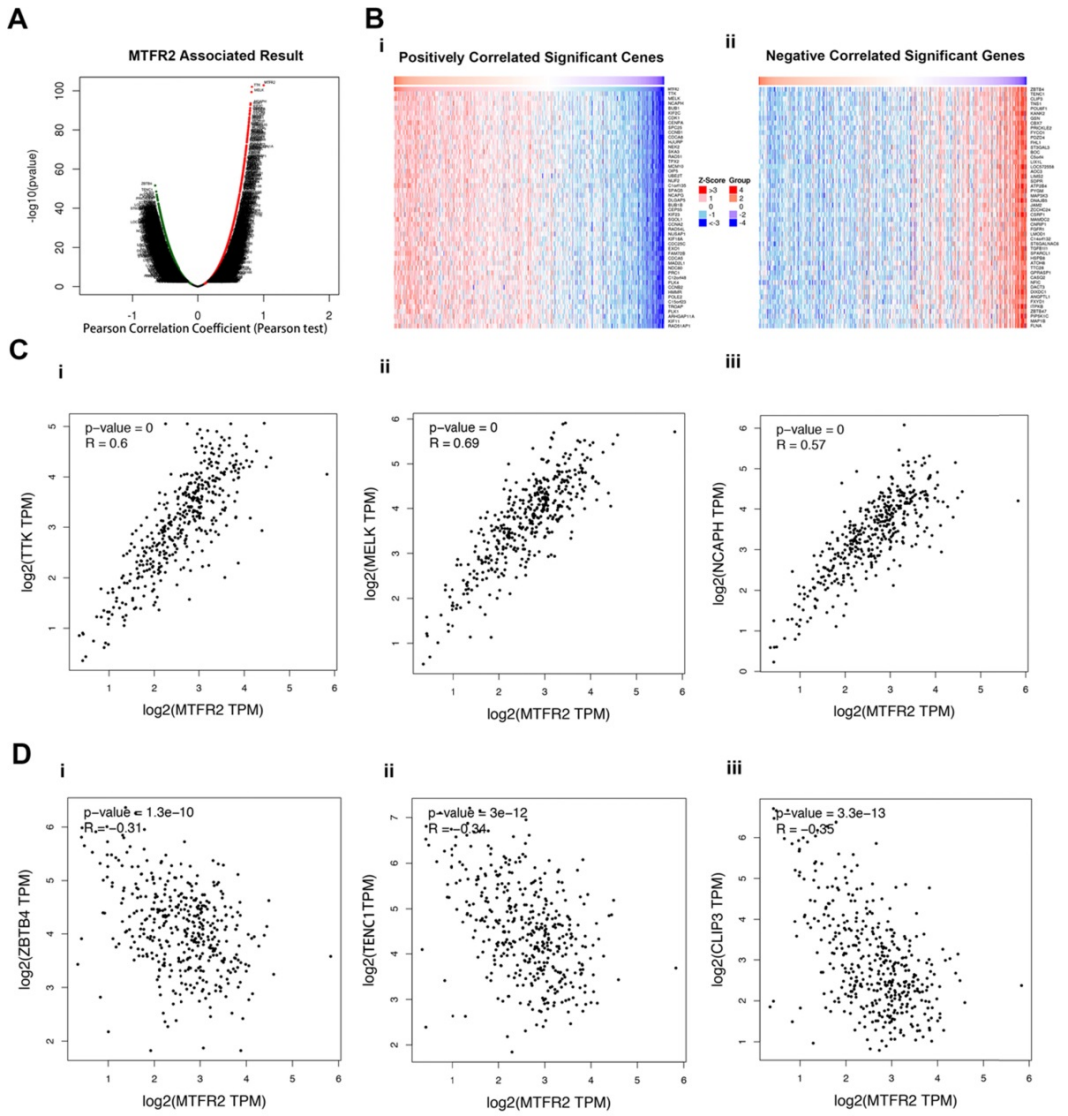
Analysis of MTFR2 co-expressed genes in GC. (A) The volcano plot of MTFR2 and its correlated genes was analyzed using LinkedOmics; (B) The heat map of MTFR2 correlated genes: (i) The top 50 positively correlated significant genes; (ii) The top 50 negatively correlated significant genes. (C) Linear regression relationships between MTFR2 and its top three positive genes was analyzed using GEPIA2: (i) MTFR2 and TTK; (ii) MTFR2 and MELK;MTFR2 and (iii) NCAPH; (D) Linear regression relationship between MTFR2 and its top three negative genes was analyzed using GEPIA2: (i) MTFR2 and ZBTB4; (ii) MTFR2 and TEBC1; (iii) MTFR2 and CLIP3.

To better understand the role of MTFR2 in the development of GC, LinkedOmics was used to analyze its co-expressed genes. As plotted in Figure 4A, 6515 genes were positively correlated with MTFR2 (red dots), while 6879 genes showed a negative correlation (green dots). The top 50 genes that were significantly positively or negatively correlated with MTFR2 are indicated with a heat map (Fig. 4B). The top three genes that were positively correlated with MTFR2 expression were TTK, MELK, and NCAPH, while ZBTB4, TENC1 and CLIP3 were the top three genes negatively correlated with MTFR2 expression. GEPIA2 was employed to verify the relationships between MTFR2 and the six co-expressed genes (Fig. 4C-D).

**Figure 5:**
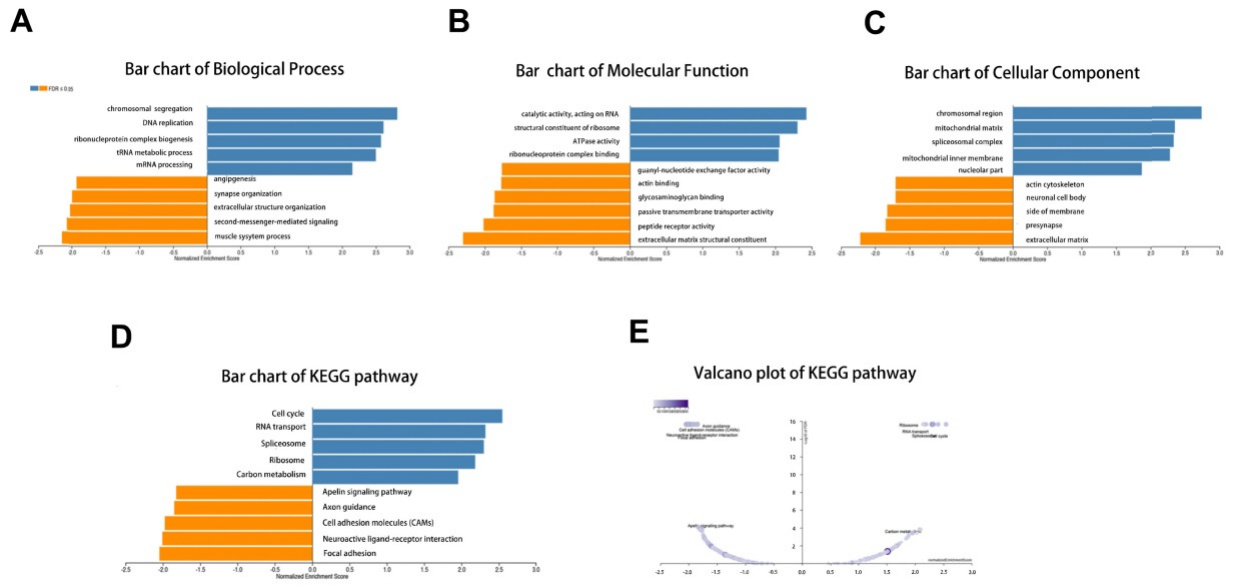
GO annotation and KEGG pathway analyses by LinkedOmics using GSEA methods: (A) Bar chart of Biological Process; (B) Bar chart of Molecular Function; (C) Bar chart of Cellular Component; (D) Bar chart of KEGG pathway; (E) Volcano plot of KEGG pathway.

Next, the enrichment functions of GO annotations and KEGG pathways were analyzed using GSEA. The main biological process identified were chromosome segregation and deoxyribonucleic acid (DNA) replication (Fig. 5A), with the molecular functions enriched in catalytic activity, acting on ribonucleic acid and ribosomal structural constituents (Fig. 5B). The top two cellular component terms were chromosomal region and mitochondrial matrix (Fig. 5C). KEGG pathway analysis revealed that the related pathways were cell cycle, spliceosome, RNA transport, and ribosome (Fig. 5D-E).

We further analyzed, using LinkedOmics, the significant kinase, miRNA, and transcription factor targets of MTFR2 in GC. CDK1, PLK1, ATM, AURKB, and ATR were the top five kinases related with MTFR2 (Tables 1 and S1). The expression of three of the top five kinase genes, CDK1, PLK1, and AURKB, were significantly higher in GC tissues and were significantly correlated with MTFR2 (Fig. S4). The potential miRNA-targets of MTFR2 were also explored, with CAGTATT (MIR-200B, MIR-200C, MIR-429); TTTGCAC (MIR-19A, MIR-19B); GTGCCAA (MIR-96); GCACCTT (MIR-18A, MIR-18B); and ATATGCA (MIR-448) identified as the top five miRNA targets associated with MTFR2 (Tables 1 and S2). Finally, the transcription factor enrichment analysis revealed that MTFR2 expression was linked to the E2F transcription factor family, which includes V$E2F_Q6, V$E2F_Q4, V$E2F1_Q6, V$E2F_Q3_01, and V$E2F_02 (Tables 1 and S3).

### MTFR2 protein interaction network and molecular docking model

**Figure 6:**
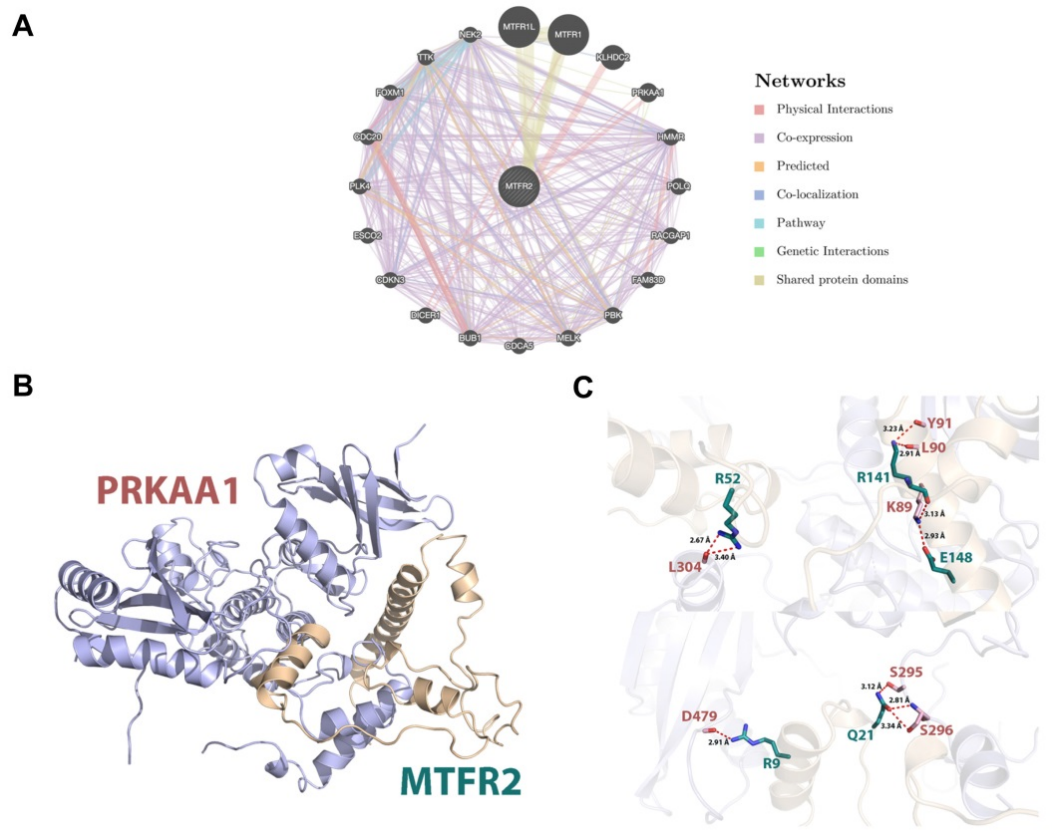
Internaction network of MTFR2 and binding mode of MTFR2 (positions 3-170) on PRKAA1 (positions 20-558). (A) Internaction network of MTFR2 constructed by GeneMANIA (lines with different colors indicate different interactive function); (B) Overall structure of MTFR2 bound to PRKAA1 in cartoon view. MTFR2 and PRKAA1 are colored in wheat, light blue respectively. Detailed interaction network between MTFR2 and PRKAA1 (C). Key residues of MTFR2 (deep teal) and PRKAA1 (pink) are displayed as sticks H-bonds are displayed in red dash lines and the distances (acceptor to donor heavy atom) of H-bonds are labeled.

Protein interactions are often required to implement biological functions and metabolic reactions. Therefore, the GeneMANIA database was used to construct an interaction network between MTFR2 and other cancer-associated proteins. We discovered that MTFR2 directly interacted with three proteins, was co-expressed with fifteen, and shared protein domains with its isoforms MTFR1 and MTFR1L (Fig. 6A). More importantly, we noticed a significant protein-protein interaction between MTFR2 and PRKAA1.

In eukaryotic cells, PRKAA1 is also a cell energy sensor. Due to the importance of MTFR2 and PRKAA1 in cell energy metabolism, we predicted the potential binding site of MTFR2 via molecular docking. As illustrated in Figure 6B, the α helixes of MTFR2 perfectly insert into a groove in PRKAA1. Hydrogen-bonding networks are critical in MTFR2-PRKAA1 binding. Five strong hydrogen-bonding interactions were detected between R52, R141, E148, R9, and Q21 of MTFR2 and L304, L90, K89, D479, and S296 of PRKAA1, respectively. In addition, R52, R141, and Q21 of MTFR2 were found to form relatively weak hydrogen bonds with PRKAA1 (Fig. 6C).

### The correlation between MTFR2 and immune infiltration level and representative immune marker genes in GC

**Figure 7:**
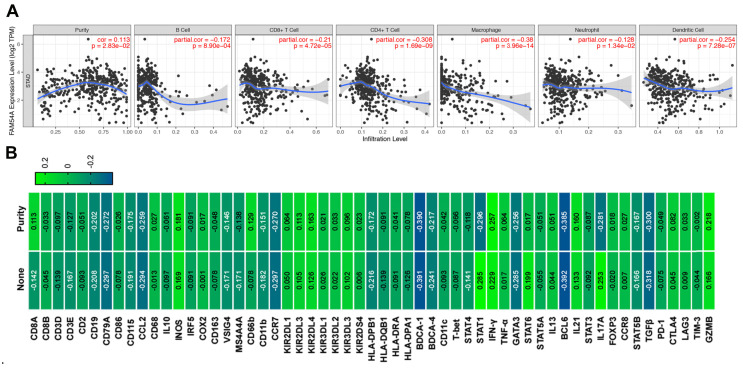
The correlation between MTFR2 expression and immune infiltration level as well as immune cell related gene markers in GC. (A) Correlation of MTFR2 expression with tumor purity and infiltrating levels of B cell, CD8+ T cell, CD4+ T cell, macrophage, neutrophil and dendritic cell in GC. (B) The correlation between MTFR2 expression and immune cells related gene markers. (None: tumor purity is not considered, it means that the tumor purity is not used to correct the results by using the partial Sperarman's correlation when performing this association analysis; Purity: tumor purity is considered, it means the tumor purity is used to correct the results when performing correlation analysis).

The immune infiltration in the tumor microenvironment is closely related to the occurrence and development of tumors. Therefore, we analyzed the correlation between MTFR2 expression and immune infiltration by TIMER database in GC. The results showed that MTFR2 was significant with tumor purity in GC (cor = 0.113, *p* =2.83E-02) (Fig. 7A). Meanwhile, MTFR2 expression was significantly correlated with the infiltration levels of B cells (patial.cor = -0.172, *p* =8.90E-04), CD8+ T cells (patial.cor = -0.21, *p* =4.72E-05), CD4+ T cells (patial.cor = -0.308, *p* = 1.69E-09), macrophages (patial.cor = -0.38, *p* = 3.96E-14), neutrophils (patial.cor = -0.128, *p* = 1.34E-02) and dendritic cells (patial.cor = -0.254, *p* = 7.28E-07) (Fig. 7A).

Next, we investigated the relevance between MTFR2 expression and the status of tumor-infiltrating immune cells based on the levels of immune marker genes in GC. The immune cells analyzed in GC tissues included CD8 + cell, B cells, tumor-associated macrophages (TAMs), neutrophils, and Dendritic cell. Moreover, different subsets of T cells, namely, T cell (general), Th1, Th2, Th17, and Tregs were also examined. The correlation between immune marker genes and MTFR2 expression with or without tumor purity was intultively displayed through Fig. 7B and Table 2. Specifically, MTFR2 expression domonstrated noticeable interaction with the markers of specific immune cells such as B cell, CD19(cor = -0.202, *p* = 7.51E-05), CD79A(cor = - 0.272, *p* =7.82E-08) ; TAM, CCL2(cor = - 0.259, *p* = 3.10E-07); Neutrophils, CCR7 (cor = - 0.270, *p* =9.58E-08); Dendritic cell, BDCA-4 (cor = -0.217, *p* =1.97E-05), Th1, STAT1 (cor = - 0.296, *p* = 4.11E-09), IFN-γ (cor = 0.257 , *p* = 3.83E-07); Th2 cell, GATA3(cor = - 0.256,* p* = 4.23E-07); Tfh cell , BCL6 (cor = - 0.385,* p* = 8.26E-15); Th17 cell, IL17A(cor = - 0.281, *p* = - 0.281);Treg cell, TGF β (cor = - 0.300, *p* = 2.70E-09) and T exhaustion cell, GZMB (cor = 0.218, *p* = 1.77E-05). These findings suggest a significant relationship between MTFR2 expression and immune infiltration.

## Discussion

Mitochondrial fusion and fission are necessary for maintaining mitochondrial homeostasis and function 32, 33. Related mitochondrial fission proteins are closely associated with cancer cell biological activities, such as cell cycle 34, cellular invasion and migration 35, and apoptosis 36. Recently, MTFR2 was reported to play an important role in promoting mitochondrial fission 11 but its biological function in GC is still unclear. In this study, MTFR2 expression levels, function, and impact on prognosis in GC was explored in depth.

Through the analysis of public databases, clinical specimens, and gastric cell lines, we found that MTFR2 is significant highly expressed in GC, and its overexpression was associated with poorer patient's prognosis. Function experiments revealed that MTFR2 down-regulation can inhibit GC cell proliferation and migration *in vitro* and in *vivo*. These results indicate that MTFR2 may be important in the occurrence and development of GC.

To clarify the potential causes of MTFR2 overexpression, we investigated MTFR2 promoter methylation levels, gene mutations, CNAs, and amplification. The results imply that elevated MTFR2 expression can, in part, result from amplification changes rather than promoter methylation and/or mutations. Notably, miRNAs and transcription factors are key epigenetic regulators of gene expression 37. Therefore, the miRNAs and transcription factors which may regulate MTFR2 were investigated by bioinformatics, and Cytoscape was used to visualize the MTFR2 regulatory network. However, further in-depth work is required to address the regulatory network of MTFR2.

The incorrect translation of oncogenes and tumor suppressor genes can promote abnormal proliferation of cancer cells 38. Kinases, transcription factors, and miRNAs are vital in these processes. MTFR2 was found to be associated with various kinases such as CDK1, PLK1, ATM, ATR, and AURKB. They mainly regulate mitosis, DNA repair signaling, genome stability, and the cell cycle in GC 39-43. Additionally, we found that the E2F transcription factor family, which regulates the cell cycle, cell differentiation, DNA damage response, and cell death, was significantly associated with MTFR2. Notably, increased E2F1 expression enhances GC cell proliferation, migration, invasion, and cell cycle progression 44, 45. Similarly, MTFR2 activates GC cell proliferation, migration, and invasion. Altogether, these results indicate that E2F1 is an important target of MTFR2 and that MTFR2 via E2F1 may regulate the biological processes of GC cells.

To thoroughly understand the biological functions of MTFR2, we identified MTFR2 co-expressed genes and verified the top three positive and negative genes using GEPIA. TTK had the strongest positive association with MTFR2 transcriptional levels. TTK, a mitotic kinase that can phosphorylate tyrosine, serine, and threonine was the top positively correlated gene of MTFR2. It affects cell proliferation by controlling the key checkpoint proteins in the process of cell division during mitosis 46, 47. TTK silencing inhibits cell proliferation, invasion, and migration in renal clear cell carcinoma 48, hepatocellular carcinoma 49, and pancreatic ductal adenocarcinoma 50. In contrast, TENC1 was one of the genes most negatively correlated with MTFR2 expression. It is a negative regulator of the protein kinase B signal transduction pathway and inhibits cell survival, proliferation, and migration 51, 52. Based on these results, MTFR2 is likely to constitute a latent determinant and contributes to the occurrence and development of GC with its correlated genes such as TTK and TENC1.

GO annotation and KEGG pathway analyses were performed to further delineate the function of MTFR2. As expected, its function and co-expressed genes were primarily related to chromosomal region, mitochondrial matrix, spliceosomal complex, chromosome segregation, mitochondrial energy metabolism, and ribosome relative activity. The cell cycle is an exquisitely tuned process; before the cell divides, the chromosomes must be replicated and precisely separated to ensure that the daughter cells contain identical copies of the genome 53, 54. Notably, pathway enrichment analysis revealed that MTFR2 co-expressed genes were significantly enriched in the cell cycle. Cell proliferation depends on an orderly cell cycle process 55. The results of our plate cloning assay confirm that MTFR2 can promote cell proliferation. Therefore, we hypothesize that MTFR2, via regulating mitochondrial energy metabolism, may modulate chromosome separation and the cell cycle, ultimately promoting cell proliferation.

The MTFR2 interaction network revealed that MTFR2 can directly interact with PRKAA1. PRKAA1, which belongs to the serine/threonine protein kinase family, is a catalytic subunit of the mammalian 5′-AMP-activated protein kinase 56. As a cell energy sensor, it regulates intracellular nutrition and energy levels via glucose and lipid metabolic pathways 57. Studies have recently reported that PRKAA1 modulates GC cell proliferation via the regulation of the c-JNK, AKT, and NF-κB signaling pathways 58, 59. Protein-protein interactions are the cornerstone of numerous biological functions. PRKAA1 may function by interacting with MTFR2. To better understand the pattern of their direct interaction, we predicted a potential binding site using a molecular docking model. This result can provide a foundation for future experimental studies.

Immunotherapy has demonstrated excellent efficacy for a variety of solid tumors and hematological malignancies 60. Immunotherapy with checkpoint-blocking antibodies targeting CTLA-4 and PD-1/PD-L1 has improved the outlook for patients with a variety of malignancies 61. However, the emergence of drug resistance prompted us to search for new immune regulation mechanisms. In our study, the level of MTFR2 expression shows negative correlation with the infiltration of B cells, CD8+ T cells, CD4+ T cells, macrophages, neutrophils and dendritic cells. We further found that MTFR2 expression showed noticeable correlation with multiple immune markers, such as CD19, CD79A, CCL2, CCR7, BDCA-4 and so on. Existing research evidence has shown that the mitochondrial dynamics of tumor cells may indirectly affect the immune response, including the activation, migration and exhaustion and apoptosis of immune cells 62. All the above findings indicate that there is a closely correlation between MTFR2 expression and immune infiltration, and it may contribute to tumor development by affecting immune infiltration in the tumor microenvironment.

There are some limitations in our study. Firstly, although we have confirmed the expression and biological function of MTFR2 in GC through experiment, the underlying mechanism of MTFR2 still lacks corresponding experimental verification. Therefore, we need to explore the mechanism of MTFR2 through further research in the future. Secondly, the correlation analysis between MTFR2 and immune infiltration is performed under the condition of adjusting tumor purification, but the sequencing data may contain information from other cell sources, which requires tissue sample confirmation.

## Conclusion

This work has effectively revealed the expression, prognostic value, potential functional networks, protein interactions and immune infiltration of MTFR2 in GC. In summary, MTFR2 is overexpressed in GC and promotes its progression. MTFR2 may be a potential prognostic marker and therapeutic target for GC patients.

## Supplementary Material

Supplementary figures and tables.

## Figures and Tables

**Table 1:** The kinases, miRNAs, and transcription factors-target networks of MTFR2 in GC.

Enriched category	Gene set	Leading edge number	NES	FDR
**Kinase Target**	Kinase_CDK1	84	2.3639	0
	Kinase_PLK1	30	2.3091	0
	Kinase_ATM	45	2.2282	0
	Kinase_AURKB	43	2.2111	0
	Kinase_ATR	31	2.186	0
**miRNA Target**	CAGTATT,MIR-200B,MIR-200C,MIR-429	132	-1.832	0
	TTTGCAC,MIR-19A,MIR-19B	165	-1.808	0
	GTGCCAA,MIR-96	99	-1.802	0
	GCACCTT,MIR-18A,MIR-18B	41	-1.784	0.0023
	ATATGCA,MIR-448	75	-1.782	0.0019
**Transcription Factor Target**	V$E2F_Q6	81	2.3869	0
	V$E2F_Q4	81	2.3704	0
	V$E2F1_Q6	82	2.293	0
	V$E2F_Q3_01	74	2.2751	0
	V$E2F_02	79	2.2607	0

**NES**: Normalized enrichment score; **FDR**: False discovery rate.

**Table 2:** Correlation analysis between MTFR2 and related genes and markers of immune cells in TIMER.

Description	Gene markers	STAD			
		None		Purity	
		Cor	*P*	Cor	*P*
**CD8+ T cell**	CD8A	-0.142	3.79E-03	0.113	1.65E-02
	CD8B	-0.045	3.65E-01	-0.033	5.27E-01
**T cell(general)**	CD3D	-0.139	4.44E-03	-0.097	6.05E-02
	CD3E	-0.167	6.37E-04	-0.127	1.30E-02
	CD2	-0.093	5.84E-02	-0.051	3.17E-01
**B cell**	CD19	-0.208	1.92E-05	-0.202	7.51E-05
	CD79A	-0.297	8.20E-10	-0.272	7.82E-08
**Monocyte**	CD86	-0.078	1.11E-01	-0.026	6.15E-01
	CD115 (CSF1R)	-0.191	9.23E-05	-0.175	6.12E-04
**TAM**	CCL2	-0.294	1.21E-09	-0.259	3.10E-07
	CD68	-0.013	7.91E-01	0.027	5.96E-01
	IL10	-0.097	4.80E-02	-0.061	2.33E-01
**M1 Macrophage**	INOS (NOS2)	0.169	5.42E-04	0.181	4.03E-04
	IRF5	-0.091	6.29E-02	-0.091	7.52E-02
	COX2(PTGS2)	-0.001	9.89E-01	0.017	7.45E-01
**M2 Macrophage**	CD163	-0.078	1.11E-01	-0.048	3.55E-01
	VSIG4	-0.171	4.62E-04	-0.146	4.47E-03
	MS4A4A	-0.171	4.78E-04	-0.138	7.03E-03
**Neutrophils**	CD66b (CEACAM8)	-0.078	9.88E-03	0.129	1.21E-02
	CD11b (ITGAM)	-0.182	1.99E-04	-0.151	3.22E-03
	CCR7	-0.297	7.95E-10	-0.270	9.58E-08
**Natural killer cell**	KIR2DL1	0.050	3.07E-01	0.064	2.16E-01
	KIR2DL3	0.105	3.28E-02	0.113	2.83E-02
	KIR2DL4	0.126	1.04E-02	0.163	1.48E-03
	KIR3DL1	0.026	6.04E-01	0.021	6.90E-01
	KIR3DL2	0.022	6.56E-01	0.033	5.18E-01
	KIR3DL3	0.102	3.78E-02	0.096	6.06E-02
	KIR2DS4	0.006	9.07E-01	0.023	6.53E-01
**Dendritic cell**	HLA-DPB1	-0.216	9.08E-06	-0.172	7.59E-04
	HLA-DQB1	-0.139	4.63E-03	-0.091	7.67E-02
	HLA-DRA	-0.091	6.33E-02	-0.041	4.26E-01
	HLA-DPA1	-0.126	1.02E-02	-0.078	1.28E-01
	BDCA-1(CD1C)	-0.391	1.26E-16	-0.390	3.29E-15
	BDCA-4(NRP1)	-0.241	7.55E-07	-0.217	1.97E-05
	CD11c (ITGAX)	-0.093	5.76E-02	-0.042	4.12E-01
**Th1**	T-bet (TBX21)	-0.087	7.55E-02	-0.066	1.98E-01
	STAT4	-0.141	4.09E-03	-0.118	2.19E-02
	STAT1	0.285	4.08E-09	-0.296	4.11E-09
	IFN-γ (IFNG)	0.229	2.51E-06	0.257	3.83E-07
	TNF-α (TNF)	0.017	7.30E-01	0.064	2.11E-01
**Th2**	GATA3	-0.285	3.87E-09	-0.256	4.23E-07
	STAT6	0.199	6.09E-01	0.017	7.46E-01
	STAT5A	-0.055	2.66E-01	-0.051	5.04E-01
	IL13	0.044	3.71E-01	0.051	3.24E-01
**Tfh**	BCL6	-0.392	1.09E-16	-0.385	8.26E-15
	IL21	0.133	6.83E-03	0.160	1.73E-03
**Th17**	STAT3	-0.092	6.21E-02	-0.087	8.90E-02
	IL17A	0.253	1.82E-07	-0.281	2.57E-08
**Treg**	FOXP3	-0.020	6.90E-01	0.018	7.29E-01
	CCR8	0.007	8.81E-01	0.027	5.96E-01
	STAT5B	-0.166	7.21E-04	-0.167	1.12E-03
	TGFβ (TGFB1)	-0.318	3.32E-11	-0.300	2.70E-09
**T cell exhaustion**	PD-1 (PDCD1)	-0.075	1.29E-01	-0.049	3.38E-01
	CTLA4	0.045	3.66E-01	0.082	1.09E-01
	LAG3	0.009	8.62E-01	0.033	5.27E-01
	TIM-3 (HAVCR2)	-0.044	3.70E-01	-0.002	9.74E-01
	GZMB	0.166	7.15E-04	0.218	1.77E-05

**STAD**: Stomach adenocarcinoma; **Cor**: Correlation coefficient; ***P***: *P value*; **TAM**: Tumor-associated macrophages; **Th1**: T-helper cell 1; **Th2**: T-helper cell 2; **Tfh**: Follicular helper T cell; **Th17**: T-helper cell 17; **Treg**: Regulatory T cell.

## Abbreviations

MTFR2: Mitochondrial fission regulator 2
GC: Gastric cancer
PRKAA1: Protein kinase adenosine-monophosphate-activated catalytic subunit alpha 1
mRNA: Messenger ribonucleic acid
IHC: Immunohistochemistry
HPA: Human protein atlas
CNAs: Copy number alterations
miRNAs: Micro ribonucleic acids
GO: Gene ontology
KEGG: Kyoto encyclopedia of genes and genomes
GSEA: Gene set enrichment analysis
OS: Overall survival
shRNA: Short hairpin ribonucleic acid
TAMs: Tumor-associated macrophages
STAD: Stomach adenocarcinoma
Cor: Correlation coefficient
*P*: *P* value
TAM: Tumor-associated macrophages
Th1: T-helper cell 1
Th2: T-helper cell 2
Tfh: Follicular helper T cell
Th17: T-helper cell 17
Treg: regulatory T cell

## References

[1] Bray F, Ferlay J, Soerjomataram I, Siegel RL, Torre LA, Jemal A (2018). Global cancer statistics 2018: GLOBOCAN estimates of incidence and mortality worldwide for 36 cancers in 185 countries. CA Cancer J Clin.

[2] Ajani JA, Lee J, Sano T, Janjigian YY, Fan D, Song S (2017). Gastric adenocarcinoma. Nat Rev Dis Primers.

[3] Shi WJ, Gao JB (2016). Molecular mechanisms of chemoresistance in gastric cancer. World journal of gastrointestinal oncology.

[4] Wallace DC (2012). Mitochondria and cancer. Nature reviews Cancer.

[5] Nunnari J, Suomalainen A (2012). Mitochondria: in sickness and in health. Cell.

[6] Calvo SE, Mootha VK (2010). The mitochondrial proteome and human disease. Annu Rev Genomics Hum Genet.

[7] Libiad M, Vitvitsky V, Bostelaar T, Bak DW, Lee HJ, Sakamoto N (2019). Hydrogen sulfide perturbs mitochondrial bioenergetics and triggers metabolic reprogramming in colon cells. The Journal of biological chemistry.

[8] Feng ST, Wang ZZ, Yuan YH, Wang XL, Sun HM, Chen NH (2020). Dynamin-related protein 1: A protein critical for mitochondrial fission, mitophagy, and neuronal death in Parkinson's disease. Pharmacol Res.

[9] Flippo KH, Strack S (2017). Mitochondrial dynamics in neuronal injury, development and plasticity. Journal of cell science.

[10] Wang J, Xie Y, Bai X, Wang N, Yu H, Deng Z (2018). Targeting dual specificity protein kinase TTK attenuates tumorigenesis of glioblastoma. Oncotarget.

[11] Monticone M, Panfoli I, Ravera S, Puglisi R, Jiang MM, Morello R (2010). The nuclear genes Mtfr1 and Dufd1 regulate mitochondrial dynamic and cellular respiration. J Cell Physiol.

[12] Lu G, Lai Y, Wang T, Lin W, Lu J, Ma Y (2019). Mitochondrial fission regulator 2 (MTFR2) promotes growth, migration, invasion and tumour progression in breast cancer cells. Aging.

[13] Lu W, Zang R, Du Y, Li X, Li H, Liu C (2020). Overexpression of MTFR2 Predicts Poor Prognosis of Breast Cancer. Cancer management and research.

[14] Rhodes DR, Yu J, Shanker K, Deshpande N, Varambally R, Ghosh D (2004). ONCOMINE: a cancer microarray database and integrated data-mining platform. Neoplasia.

[15] Rhodes DR, Kalyana-Sundaram S, Mahavisno V, Varambally R, Yu J, Briggs BB (2007). Oncomine 3.0: genes, pathways, and networks in a collection of 18,000 cancer gene expression profiles. Neoplasia.

[16] Tang Z, Li C, Kang B, Gao G, Li C, Zhang Z (2017). GEPIA: a web server for cancer and normal gene expression profiling and interactive analyses. Nucleic acids research.

[17] Tang Z, Kang B, Li C, Chen T, Zhang Z (2019). GEPIA2: an enhanced web server for large-scale expression profiling and interactive analysis. Nucleic acids research.

[18] Shin G, Kang TW, Yang S, Baek SJ, Jeong YS, Kim SY (2011). GENT: gene expression database of normal and tumor tissues. Cancer Inform.

[19] Ezkurdia I, Calvo E, Del Pozo A, Vazquez J, Valencia A, Tress ML (2015). The potential clinical impact of the release of two drafts of the human proteome. Expert Rev Proteomics.

[20] Thul PJ, Akesson L, Wiking M, Mahdessian D, Geladaki A, Ait Blal H (2017). A subcellular map of the human proteome. Science.

[21] Uhlen M, Zhang C, Lee S, Sjostedt E, Fagerberg L, Bidkhori G (2017). A pathology atlas of the human cancer transcriptome. Science.

[22] Szasz AM, Lanczky A, Nagy A, Forster S, Hark K, Green JE (2016). Cross-validation of survival associated biomarkers in gastric cancer using transcriptomic data of 1,065 patients. Oncotarget.

[23] Gao J, Aksoy BA, Dogrusoz U, Dresdner G, Gross B, Sumer SO (2013). Integrative analysis of complex cancer genomics and clinical profiles using the cBioPortal. Sci Signal.

[24] Cerami E, Gao J, Dogrusoz U, Gross BE, Sumer SO, Aksoy BA (2012). The cBio cancer genomics portal: an open platform for exploring multidimensional cancer genomics data. Cancer Discov.

[25] Agarwal V, Bell GW, Nam JW, Bartel DP (2015). Predicting effective microRNA target sites in mammalian mRNAs. eLife.

[26] Farré D, Roset R, Huerta M, Adsuara JE, Roselló L, Albà MM (2003). Identification of patterns in biological sequences at the ALGGEN server: PROMO and MALGEN. Nucleic acids research.

[27] Shannon P, Markiel A, Ozier O, Baliga NS, Wang JT, Ramage D (2003). Cytoscape: a software environment for integrated models of biomolecular interaction networks. Genome research.

[28] Vasaikar SV, Straub P, Wang J, Zhang B (2018). LinkedOmics: analyzing multi-omics data within and across 32 cancer types. Nucleic acids research.

[29] Warde-Farley D, Donaldson SL, Comes O, Zuberi K, Badrawi R, Chao P (2010). The GeneMANIA prediction server: biological network integration for gene prioritization and predicting gene function. Nucleic acids research.

[30] Li T, Fan J, Wang B, Traugh N, Chen Q, Liu J TIMER: A Web Server for Comprehensive Analysis of Tumor-Infiltrating Immune Cells. 2017; 77: e108-e10.

[31] Wang G, Zhong WC, Bi YH, Tao SY, Zhu H, Zhu HX (2019). The Prognosis Of Peroxiredoxin Family In Breast Cancer. Cancer management and research.

[32] Lin S, Hoffmann K, Gao C, Petrulionis M, Herr I, Schemmer P (2017). Melatonin promotes sorafenib-induced apoptosis through synergistic activation of JNK/c-jun pathway in human hepatocellular carcinoma. J Pineal Res.

[33] Ruan W, Lim HH, Surana U (2018). Mapping Mitotic Death: Functional Integration of Mitochondria, Spindle Assembly Checkpoint and Apoptosis. Front Cell Dev Biol.

[34] Wen H, Zhou S, Song J (2019). Induction of apoptosis by magnolol via the mitochondrial pathway and cell cycle arrest in renal carcinoma cells. Biochem Biophys Res Commun.

[35] Lima AR, Santos L, Correia M, Soares P, Sobrinho-Simoes M, Melo M (2018). Dynamin-Related Protein 1 at the Crossroads of Cancer. Genes (Basel).

[36] Sheridan C, Martin SJ (2010). Mitochondrial fission/fusion dynamics and apoptosis. Mitochondrion.

[37] Jiang W, Mitra R, Lin CC, Wang Q, Cheng F, Zhao Z (2016). Systematic dissection of dysregulated transcription factor-miRNA feed-forward loops across tumor types. Briefings in bioinformatics.

[38] Smit KN, Chang J, Derks K, Vaarwater J, Brands T, Verdijk RM (2019). Aberrant MicroRNA Expression and Its Implications for Uveal Melanoma Metastasis. Cancers.

[39] Kim SJ, Lee HW, Baek JH, Cho YH, Kang HG, Jeong JS (2016). Activation of nuclear PTEN by inhibition of Notch signaling induces G2/M cell cycle arrest in gastric cancer. Oncogene.

[40] Lin X, Chen D, Zhang C, Zhang X, Li Z, Dong B (2018). Augmented antitumor activity by olaparib plus AZD1775 in gastric cancer through disrupting DNA damage repair pathways and DNA damage checkpoint. Journal of experimental & clinical cancer research: CR.

[41] Subhash VV, Tan SH, Yeo MS, Yan FL, Peethala PC, Liem N (2016). ATM Expression Predicts Veliparib and Irinotecan Sensitivity in Gastric Cancer by Mediating P53-Independent Regulation of Cell Cycle and Apoptosis. Molecular cancer therapeutics.

[42] Nie M, Wang Y, Yu Z, Li X, Deng Y, Wang Y (2020). AURKB promotes gastric cancer progression via activation of CCND1 expression. Aging.

[43] Tang Z, Li L, Tang Y, Xie D, Wu K, Wei W (2018). CDK2 positively regulates aerobic glycolysis by suppressing SIRT5 in gastric cancer. Cancer science.

[44] Yan LH, Wei WY, Cao WL, Zhang XS, Xie YB, Xiao Q (2014). Overexpression of E2F1 in human gastric carcinoma is involved in anti-cancer drug resistance. BMC cancer.

[45] Xu TP, Wang YF, Xiong WL, Ma P, Wang WY, Chen WM (2017). E2F1 induces TINCR transcriptional activity and accelerates gastric cancer progression via activation of TINCR/STAU1/CDKN2B signaling axis. Cell death & disease.

[46] Mills GB, Schmandt R, McGill M, Amendola A, Hill M, Jacobs K (1992). Expression of TTK, a novel human protein kinase, is associated with cell proliferation. The Journal of biological chemistry.

[47] Sliedrecht T, Zhang C, Shokat KM, Kops GJ (2010). Chemical genetic inhibition of Mps1 in stable human cell lines reveals novel aspects of Mps1 function in mitosis. PLoS One.

[48] Woodford MR, Truman AW, Dunn DM, Jensen SM, Cotran R, Bullard R (2016). Mps1 Mediated Phosphorylation of Hsp90 Confers Renal Cell Carcinoma Sensitivity and Selectivity to Hsp90 Inhibitors. Cell Rep.

[49] Liu X, Liao W, Yuan Q, Ou Y, Huang J (2015). TTK activates Akt and promotes proliferation and migration of hepatocellular carcinoma cells. Oncotarget.

[50] Slee RB, Grimes BR, Bansal R, Gore J, Blackburn C, Brown L (2014). Selective inhibition of pancreatic ductal adenocarcinoma cell growth by the mitotic MPS1 kinase inhibitor NMS-P715. Molecular cancer therapeutics.

[51] Chen H, Duncan IC, Bozorgchami H, Lo SH (2002). Tensin1 and a previously undocumented family member, tensin2, positively regulate cell migration. Proc Natl Acad Sci USA.

[52] Hafizi S, Ibraimi F, Dahlback B (2005). C1-TEN is a negative regulator of the Akt/PKB signal transduction pathway and inhibits cell survival, proliferation, and migration. Faseb j.

[53] Zeeshan M, Pandey R, Ferguson DJP, Tromer EC, Markus R, Abel S (2020). Real-time dynamics of Plasmodium NDC80 reveals unusual modes of chromosome segregation during parasite proliferation. Journal of cell science.

[54] Seaton DD, Krishnan J (2016). Model-Based Analysis of Cell Cycle Responses to Dynamically Changing Environments. PLoS computational biology.

[55] Zhang H, Wang Z, Xie L, Zhang Y, Deng T, Li J (2018). Molecular Recognition and In-Vitro-Targeted Inhibition of Renal Cell Carcinoma Using a DNA Aptamer. Molecular therapy Nucleic acids.

[56] Krishan S, Richardson DR, Sahni S (2014). Gene of the month. AMP kinase (PRKAA1). Journal of clinical pathology.

[57] Gleason CE, Lu D, Witters LA, Newgard CB, Birnbaum MJ (2007). The role of AMPK and mTOR in nutrient sensing in pancreatic beta-cells. The Journal of biological chemistry.

[58] Zhang Y, Zhou X, Cheng L, Wang X, Zhang Q, Zhang Y (2020). PRKAA1 Promotes Proliferation and Inhibits Apoptosis of Gastric Cancer Cells Through Activating JNK1 and Akt Pathways. Oncology research.

[59] Zhang Y, Zhou X, Zhang Q, Zhang Y, Wang X, Cheng L (2019). Involvement of NF-κB signaling pathway in the regulation of PRKAA1-mediated tumorigenesis in gastric cancer. Artificial cells, nanomedicine, and biotechnology.

[60] Chen P, Hsu W, Han J, Xia Y, DePinho R (2021). Cancer Stemness Meets Immunity: From Mechanism to Therapy. Cell Rep.

[61] Patel S, Minn A (2018). Combination Cancer Therapy with Immune Checkpoint Blockade: Mechanisms and Strategies. Immunity.

[62] Simula L, Nazio F, Campello S (2017). The mitochondrial dynamics in cancer and immune-surveillance. Seminars in Cancer Biology.

