# Supplementary material for: MTFR2, A Potential Biomarker for Prognosis and Immune Infiltrates, Promotes Progression of Gastric Cancer Based on Bioinformatics Analysis and Experiments
Source: J Cancer. 2021 Apr 26;12(12):3611–25. doi: 10.7150/jca.58158 (PMC8120185; doi:10.7150/jca.58158)
Supplement: Supplementary file 1 — Supplementary figures and tables. [file jcav12p3611s1.pdf]

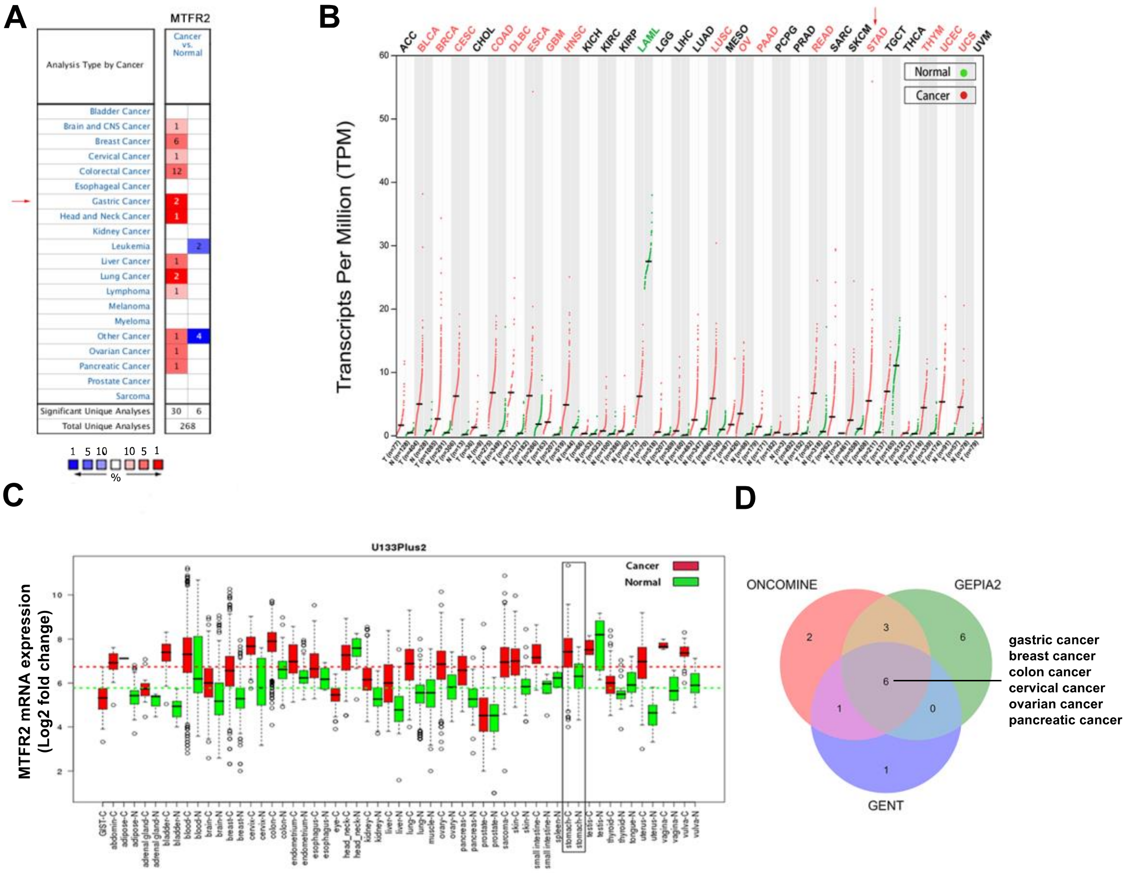

Figure S1. MTFR2 mRNA expression in various cancers. (A) The mRNA expression levels of MTFR2 in tumors and adjacent normal tissues using the ONCOMINE database. The color code indicates the best Gene code percentile(%) for the analysis. The filter condition was set as: p value<0.0001, fold change>2, gene rank: 10%, data type: mRNA. (B) The MTFR2 expression profiles across all tumors and corresponding normal tissues were showed by dot plot (GEPIA2), each dots represent expression of samples. (C) Expression pattern of MTFR2 mRNA between tumor and adjacent normal tissue which data were retrieved from the GENT (Gene Expression across Normal and Tumor tissue) in U133plus2 platform. (D) Analyses from three databases revealed that MTFR2 expresion level were all significantly up-regulated in six types of cancers (gastric cancer, breast cancer, colon cancer, cervical cancer, ovarian cancer, pancreatic cancer).

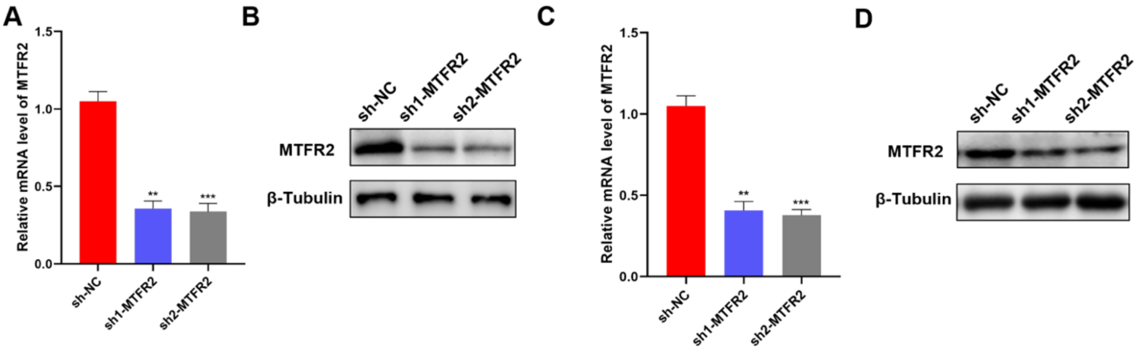

Figure S2. The Efficiency of MTFR2 knockdown was confirmed by RT-qPCR and western blot in MGC803 and MKN45 cells. (A-B) the relevant mRNA and protein expression of MTFR2 in sh-NC,sh1-MTFR2 and sh2-MTFR2 in MGC803; (C-D) the relevant mRNA and protein expression of MTFR2 in sh-NC,sh1-MTFR2 and sh2-MTFR2 in MKN45 (**P < 0.01, ***P < 0.001).

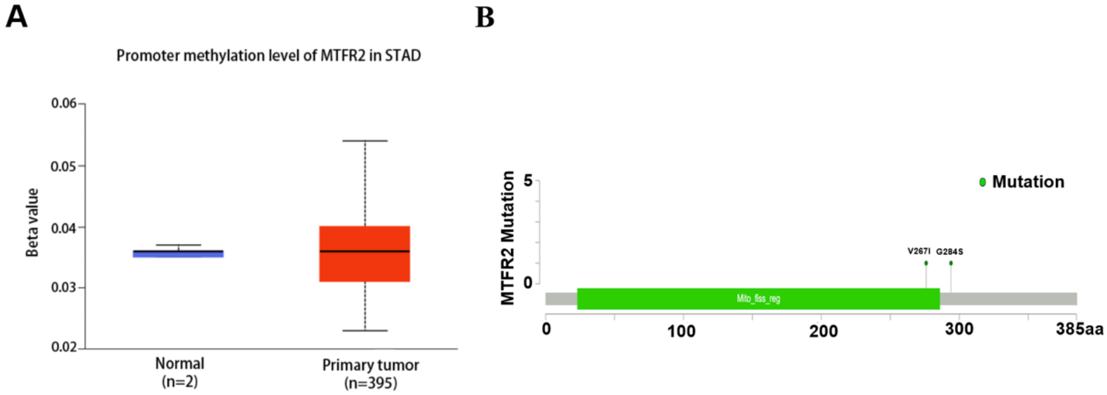

Figure S3. The promoter methylation level and mutation site of MTFR2 in GC. (A) The promoter methylation level of MTFR2 in GC; (B) The mutation site of MTFR2 in GC.

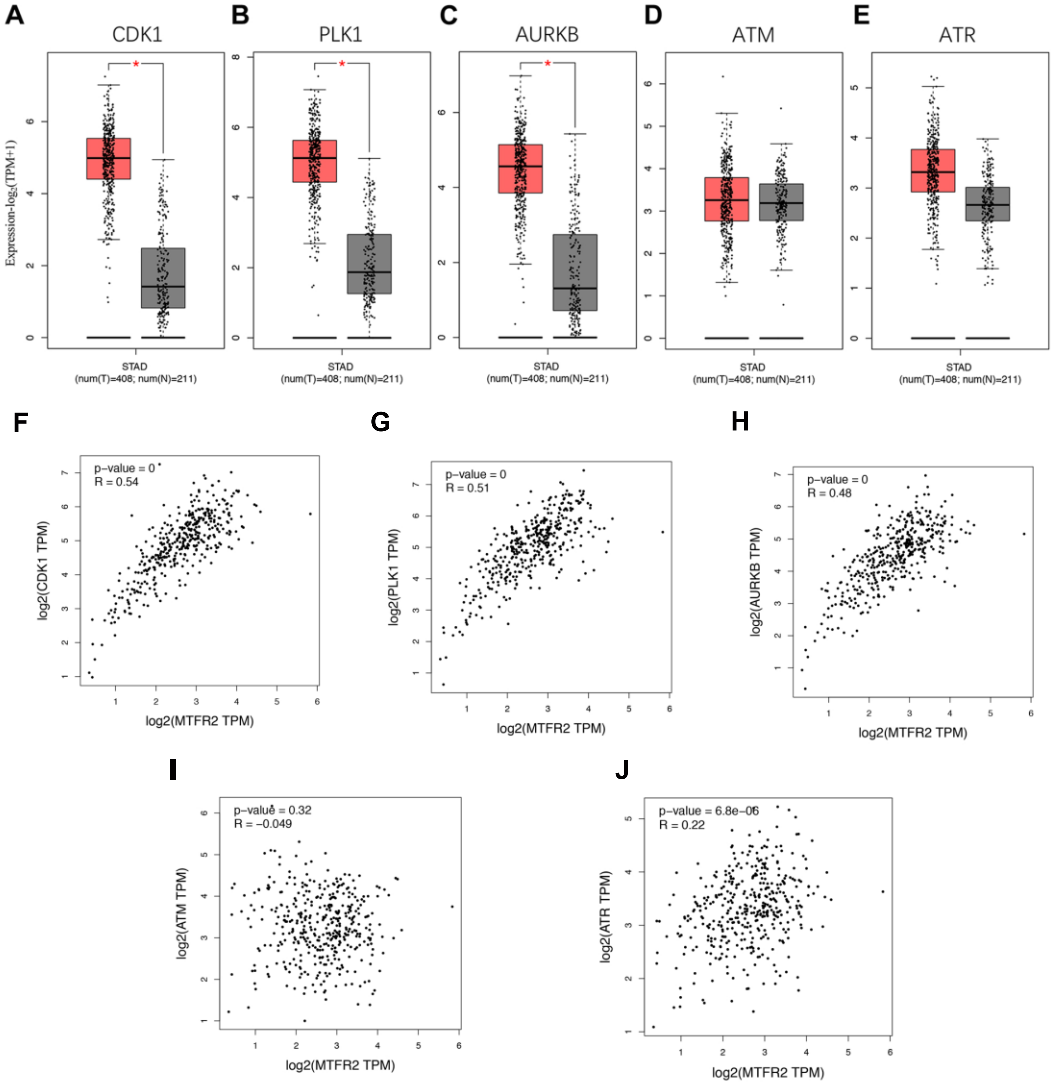

Figure S4. The expression level of five kinases and their correlation with MTFR2 in GC tissues.(A) The expression level of CDK1 in GC; (B) The expression level of PLK1 in GC; (C) The expression level of ATM in GC; (D) The expression level of AURKB in GC; (E) The expression level of ATR in GC; (F-J) The correlation between MTFR2 and CDK1, PLK1, AURKB, ATM, ATR in GC tissues.

Table S1: The kinases-target networks of MTFR2 co-expressed genes.

Gene Set	Description	Leading Edge Number	NES	*P* Value	FDR
Kinase_CDK1	cyclin dependent kinase 1	84	2.3639	0	0
Kinase_PLK1	polo like kinase 1	30	2.3091	0	0
Kinase_ATM	ATM serine/threonine kinase	45	2.2282	0	0
Kinase_AURKB	aurora kinase B	43	2.2111	0	0
Kinase_ATR	ATR serine/threonine kinase	31	2.186	0	0
Kinase_CHEK1	checkpoint kinase 1	42	2.1624	0	0
Kinase_CDK2	cyclin dependent kinase 2	87	2.0922	0	0
Kinase_CHEK2	checkpoint kinase 2	11	1.9457	0	0
Kinase_AURKA	aurora kinase A	14	1.8672	0	0.0008
Kinase_PLK3	polo like kinase 3	10	1.8596	0	0.0009
Kinase_CSNK2A1	casein kinase 2 alpha 1	72	1.8058	0	0.0022
Kinase_TTK	TTK protein kinase	8	1.728	0.0048	0.0088
Kinase_WEE1	WEE1 G2 checkpoint kinase	5	1.6605	0	0.029
Kinase_NEK2	NIMA related kinase 2	5	1.6037	0.0095	0.061
Kinase_PRKDC	protein kinase, DNA-activated, catalytic polypeptide	18	1.5868	0.0095	0.072
Kinase_LATS1	large tumor suppressor kinase 1	1	1.5855	0	0.0696
Kinase_BUB1	BUB1 mitotic checkpoint serine/threonine kinase	3	1.5746	0.0042	0.0728
Kinase_RPS6KA4	ribosomal protein S6 kinase A4	10	1.5742	0	0.0688
Kinase_PKMYT1	protein kinase, membrane associated tyrosine/threonine 1	3	1.5714	0	0.0675
Kinase_PKN2	protein kinase N2	3	1.5661	0.0143	0.0682
Kinase_MKNK1	MAP kinase interacting serine/threonine kinase 1	4	1.5251	0.0138	0.1038
Kinase_NEK1	NIMA related kinase 1	3	1.5189	0.009	0.1068
Kinase_CAMKK1	calcium/calmodulin dependent protein kinase kinase 1	4	-1.387	0.0769	0.3084
Kinase_PIK3CA	phosphatidylinositol-4,5-bisphosphat e 3-kinase catalytic subunit alpha	3	-1.397	0.0777	0.2914
Kinase_GRK5	G protein-coupled receptor kinase 5	3	-1.399	0.0468	0.297
Kinase_CSNK1A1L	casein kinase 1 alpha 1 like	3	-1.415	0.0601	0.2902

Kinase_NTRK1	neurotrophic receptor tyrosine kinase 1	9	-1.419	0.0483	0.2899
Kinase_PRKCA	protein kinase C alpha	54	-1.422	0	0.2922
Kinase_CSNK1G3	casein kinase 1 gamma 3	3	-1.431	0.0178	0.2763
Kinase_NTRK2	neurotrophic receptor tyrosine kinase 2	9	-1.434	0.0526	0.282
Kinase_SGK1	serum/glucocorticoid regulated kinase 1	6	-1.448	0.047	0.2579
Kinase_CSNK1G2	casein kinase 1 gamma 2	4	-1.46	0.0254	0.2356
Kinase_ROCK1	Rho associated coiled-coil containing protein kinase 1	14	-1.463	0.0229	0.241
Kinase_ADRBK1	G protein-coupled receptor kinase 2	9	-1.478	0.0162	0.2167
Kinase_HCK	HCK proto-oncogene, Src family tyrosine kinase	10	-1.486	0.039	0.2104
Kinase_MYLK2	myosin light chain kinase 2	3	-1.498	0.0214	0.1937
Kinase_PRKACA	protein kinase cAMP-activated catalytic subunit alpha	97	-1.498	0	0.2131
Kinase_PAK6	p21 (RAC1) activated kinase 6	2	-1.506	0.013	0.2101
Kinase_MARK2	microtubule affinity regulating kinase 2	10	-1.571	0.0103	0.0886
Kinase_PRKG1	protein kinase, cGMP-dependent, type I	10	-1.588	0.0068	0.0738
Kinase_CDK5	cyclin dependent kinase 5	25	-1.613	0	0.0593
Kinase_PRKACG	protein kinase cAMP-activated catalytic subunit gamma	26	-1.658	0.003	0.0289
Kinase_PRKACB	protein kinase cAMP-activated catalytic subunit beta	27	-1.687	0	0.0199
Kinase_PRKX	protein kinase, X-linked	27	-1.693	0	0.0223
Kinase_FYN	FYN proto-oncogene, Src family tyrosine kinase	22	-1.693	0	0.0335
Kinase_DAPK3	death associated protein kinase 3	5	-1.764	0	0.0125

Table S2: The kinases-target networks of MTFR2 co-expressed genes.

Gene Set	Leading Edge Number	NES	*P* Value	FDR
CAGTATT,MIR-200B,MIR-200C,MIR-429	132	-1.832	0	0
TTTGCAC,MIR-19A,MIR-19B	165	-1.808	0	0
GTGCCAA,MIR-96	99	-1.802	0	0
GCACCTT,MIR-18A,MIR-18B	41	-1.784	0	0.0023
ATATGCA,MIR-448	75	-1.782	0	0.0019
GTGCAAA,MIR-507	42	-1.766	0	0.0021
GACAATC,MIR-219	45	-1.748	0	0.0036
GTGTCAA,MIR-514	26	-1.727	0	0.0043
CAGCACT,MIR-512-3P	61	-1.723	0	0.0049
GTATTAT,MIR-369-3P	63	-1.715	0	0.005
ACACTGG,MIR-199A,MIR-199B	46	-1.714	0	0.0051
TGTGTGA,MIR-377	59	-1.7	0	0.0052
CTTTGCA,MIR-527	85	-1.694	0	0.0053
GTGCAAT,MIR-25,MIR-32,MIR-92,MIR-363, MIR-367	98	-1.692	0	0.0049
ATGTAGC,MIR-221,MIR-222	42	-1.675	0	0.0065
GCAAAAA,MIR-129	48	-1.671	0	0.0062
ATGTACA,MIR-493	99	-1.669	0	0.0062
CCAGGGG,MIR-331	26	-1.666	0.0032	0.0059
CCTGTGA,MIR-513	35	-1.662	0	0.0061
TGTATGA,MIR-485-3P	43	-1.659	0	0.0061
GAGCCTG,MIR-484	32	-1.65	0	0.0064
CCCAGAG,MIR-326	41	-1.648	0	0.0062
GGTGTGT,MIR-329	46	-1.646	0	0.0062
TCCAGAG,MIR-518C	51	-1.644	0	0.0061
ACATATC,MIR-190	19	-1.641	0.0033	0.0061
GTAGGCA,MIR-189	7	0.6637	0.9476	0.9827
TGCACGA,MIR-517A,MIR-517C	3	0.6768	0.9063	1
AGTGCGT,MIR-521	1	0.7385	0.76	1
AACGGTT,MIR-451	2	0.8162	0.7108	1
AGTCTAG,MIR-151	3	0.8394	0.6996	1

AGCGCAG,MIR-191	2	0.8901	0.6172	1
CCAGGTT,MIR-490	8	1.0829	0.3036	1
ACCGAGC,MIR-423	3	1.1509	0.34	1

Table S3: The transcription factor -target networks of MTFR2 co-expressed genes.

Gene Set	Leading Edge Number	NES	*P* Value	FDR
V$E2F_Q6	81	2.3869	0	0
V$E2F_Q4	81	2.3704	0	0
V$E2F1_Q6	82	2.293	0	0
V$E2F_Q3_01	74	2.2751	0	0
V$E2F_02	79	2.2607	0	0
V$E2F1_Q4_01	72	2.2586	0	0
V$E2F1DP1_01	79	2.2433	0	0
V$E2F1DP2_01	79	2.2433	0	0
V$E2F4DP2_01	79	2.2433	0	0
V$E2F_03	66	2.2332	0	0
V$E2F_Q4_01	77	2.2192	0	0
V$E2F1DP1RB_01	75	2.2148	0	0
SGCGSSAAA_V$E2F1DP2_01	66	2.2121	0	0
V$E2F4DP1_01	87	2.2078	0	0
V$E2F_Q6_01	75	2.1977	0	0
V$E2F_Q3	63	2.179	0	0
V$E2F1_Q6_01	81	2.1762	0	0
V$E2F1_Q3	76	2.1745	0	0
V$E2F1_Q4	62	1.9269	0	0
SCGGAAGY_V$ELK1_02	374	1.9227	0	0
GGAANCGGAANY_UNKNOWN	39	1.8028	0	0.0002
V$TAL1BETAE47_01	85	-1.83	0	0
V$CRX_Q4	62	-1.837	0	0
V$MEF2_01	41	-1.838	0	0
V$MYOGENIN_Q6	82	-1.839	0	0
V$CHX10_01	75	-1.841	0	0
V$RSRFC4_Q2	77	-1.854	0	0

V$NKX62_Q2	61	-1.857	0	0
CAGNYGKNAAA_UNKNOWN	31	-1.866	0	0
AAANWWTGC_UNKNOWN	66	-1.872	0	0
V$TAL1BETAITF2_01	74	-1.878	0	0
V$SRY_02	105	-1.883	0	0
V$RSRFC4_01	84	-1.889	0	0
V$SRF_01	20	-1.898	0	0
CTGCAGY_UNKNOWN	223	-1.903	0	0
V$AFP1_Q6	73	-1.907	0	0
V$OCT1_04	83	-1.927	0	0
V$CDC5_01	69	-1.928	0	0
V$HMEF2_Q6	51	-1.928	0	0
V$MEF2_02	99	-1.952	0	0
V$LHX3_01	65	-1.962	0	0
V$RP58_01	87	-1.98	0	0
V$SRF_Q6	97	-2.078	0	0
V$SRF_C	85	-2.127	0	0
